# Supplementary material for: Effectiveness of the Mindfulness-Based Social–Emotional Growth (MSEG) Program in Enhancing Mental Health of Elementary School Students in Korea
Source: Behav Sci (Basel). 2025 Mar 5;15(3):315. doi: 10.3390/bs15030315 (PMC11939644; doi:10.3390/bs15030315)
Supplement: Supplementary file 1 [file behavsci-15-00315-s001.zip › behavsci-3472122-supplementary.pdf]

MSEG Program Content and Activities

Each weekly session centered on a specific theme and incorporated distinct mindfulness-based activities tailored to children's developmental levels. The program content progressively expanded from fundamental self-awareness practices to broader social and global themes, ensuring a cohesive learning trajectory. Key activities and focuses of the 12 sessions were as follows:

1. **Session S1 – Healthy Brain and Mindfulness:** This introductory session familiarizes students with basic mindfulness concepts and the idea of neuroplasticity. Students engage in a simple, age-appropriate demonstration of how the brain can change (e.g., noting how focusing attention improves with practice) and participate in a brief guided mindfulness exercise (such as a breathing or listening meditation) to experience mindful awareness. Additionally, the class establishes "ground rules" or a group pledge for a safe, respectful learning environment, laying the groundwork for open-mindedness and curiosity.

2. **Session S2 – Matters Important to Me:** Students identify and reflect on what is important for their body and mind, building self-awareness of personal values and needs. This session often uses a guided reflection activity inspired by the ACT Matrix (Acceptance and Commitment Training framework), in which children explore who and what matters to them and how they can care for these priorities. Through discussions and a simple charting or drawing exercise, students practice articulating personally meaningful aspects of their lives, strengthening their connection to their values and enhancing engagement in the program.

3. **Session S3 – Mindfulness through Movements of Five Forest Animal Friends:** This session introduces mindful movement in a playful and engaging manner. Students practice yoga-like poses and movements inspired by five forest animals (such as a deer, crane, tiger, bear, and monkey), enhancing their awareness of bodily sensations and improving concentration. The activity is structured as an interactive game in which each animal's movement is accompanied by a discussion of its positive qualities. Students are encouraged to observe and compliment each other's strengths during the exercise, fostering a supportive group atmosphere. This child-friendly movement activity not only enhances physical awareness and balance but also cultivates curiosity and kindness as core mindfulness principles.

4. **Session S4 – Mindfulness in Caring for the Body:** In this session, students practice accepting their bodies as they are and learn relaxation techniques through playful activity. Activities focus on awakening the five senses and tuning into bodily signals. For example, children might engage in a tactile or stretching exercise and then share how their body feels. By the end of the session, students practice a brief body-scan or progressive muscle relaxation game activity, promoting comfort with their bodies and an understanding of the value of mindful acceptance.

5. **Session S5 – Practicing Mindful Breathing:** Students learn and practice mindful breathing techniques as a foundation for self-regulation. The instructor introduces the distinction between long, deep breaths and short, quick breaths, often using an interactive activity (for instance, having children take a deep breath and a shallow panting breath to compare how each feels). Students observe how breathing rhythm influences their heart rate or feelings, thereby linking breath to emotional regulation. Through guided breathing exercises (such as belly breathing with a hand on the stomach or counting breaths), children experience how focusing on the breath can bring calm and improve concentration. This session solidifies concentration skills and gives students a practical tool (mindful breathing) to manage stress and navigate difficult emotions.

6. **Session S6 – Mindful Eating:** In this highly experiential session, students practice mindfulness through eating, engaging all five senses to fully experience a simple food item. Each child is given a small piece of food (adapted to be age-appropriate: for example, younger children might receive a tangerine, while older children work with a dried persimmon). Guided by the instructor, they slowly explore the look, texture, smell, and taste of the food, noticing sensations and reactions in detail. This slow, deliberate eating practice cultivates awareness of the present moment and self-management of impulses. It also introduces an element of gratitude – older students discuss the origins of the food and the effort involved in its production, fostering appreciation for others' contributions (younger students focus more on the immediate sensory experience). By the end of the exercise, many students report a deeper enjoyment of their food and an increased sense of gratitude, illustrating the transformative power of mindfulness in everyday activities.

7. **Session S7 – Mindfulness of Emotions:** This session shifts the focus to emotional awareness. Students reflect on emotions they have recently experienced in daily life and learn to identify and label these feelings mindfully. The instructor might use emotion cards or a feelings chart to help children recognize different emotions and associate them with corresponding facial expressions or bodily sensations. Students are guided to connect the dots between a situation, the thoughts about it, and the emotion that arises (for example, "*When I lost my toy (situation), I thought I would never find it (thought), and I felt sad (emotion)*"). They then practice a brief mindfulness exercise, such as closing their eyes and noticing how emotions like anger or happiness feel in the body, without immediately reacting.

   To build resilience, this session also introduces the idea of "savoring" positive experiences – students recall a pleasant moment and pay attention to the good feelings it brings, which reinforcing their ability to experience and remember positive emotions. Through these activities, this session fopster both self-awareness and basic self-management skills regarding emotions.

8. **Session S8 – Mindfulness of Gratitude and Kindness:** In this session, students cultivate compassion and kindness toward themselves and others, bridging self-awareness with social awareness. Activities are designed to help students appreciate both themselves and their peers while practicing self-care. For example, one activity involves students listing or drawing things they are grateful for in their lives, then sharing one with the class to reinforce a positive mindset. Another key exercise is a self-compassion practice in which children discover their own "comforting touch"—a simple physical gesture, such as placing a hand on their heart or giving themselves a gentle hug—that can provide soothing comfort when they feel upset.

   Additionally, students engage in role-playing exercises where they offer kind words to both a classmate and themselves, emphasizing that kindness should be directed inward as well as outward. By the end of the session, students recognize that gratitude and kindness can significantly enhance their mood and strengthen relationships, aligning with the session's goal of nurturing compassion.

9. **Session S9 – Mindfulness of Considering Others' Perspectives:** This session further develops social awareness by actively engaging students in perspective-taking. The class explores how individuals may think or feel differently about the same situation. A key activity involves role-playing or storytelling from multiple perspectives. For example, younger children might listen to a short story or watch a brief animation about two characters (e.g., forest animal friends) and then take turns acting out how each character perceives the events. Older students may be presented with a real-life scenario and asked to imagine it

from the perspective of a friend or classmate, comparing it to their own viewpoint.

Following each role-play, the group reflects on how their thoughts and emotions shifted based on the perspective they assumed. This immersive approach helps students recognize that everyone has unique thoughts, feelings, and needs—a foundational lesson in empathy. These activities strengthen students' ability to step outside their own point of view, an essential social-emotional skill.

10. **Session S10 – Mindfulness in Social Interaction:** Building on the previous session, Session 10 emphasizes mindful engagement and kindness in real interpersonal situations, strengthening students' relationship skills. Students participate in a cooperative group activity that fosters teamwork and kindness—such as a collaborative game or a group challenge where success depends on each member being attentive and supportive. During the activity, instructors prompt students to practice mindful communication skills, including active listening and emotional awareness (e.g., noticing if they feel impatient or appreciative while working with others).

    Following the activity, the class reflects on how acting with kindness and understanding influenced their experience. Another component may involve students planning a small act of kindness for someone in their life and sharing their plan with the group, reinforcing the habit of considering others. Through these exercises, students gain firsthand experience of how mindfulness and empathy can enhance social interactions and foster positive connections with peers.

11. **Session S11 – Mindfulness in Connecting with Nature:** In this session, the scope of mindfulness expands to include students' relationship with the natural world, integrating social awareness with responsible decision-making. The session often begins with a discussion contrasting "Screen Time" and "Green Time" (time spent on electronic devices versus time spent outdoors in nature) to highlight the effects of each on well-being. Students may watch a short video or listen to a story illustrating how time in nature can calm the mind and improve mood (for example, a "nature heals" video demonstrating the benefits of greenery and outdoor play).

    The main activity involves a nature-focused mindfulness exercise. If possible, the class goes outside to a school garden or playground; if indoors, the instructor provides natural objects such as plants, leaves, or stones. Students are invited to quietly observe or handle these natural elements, noticing colors, textures, sounds, and scents. Each student selects a natural object that resonates with them or represents them in some way and shares why they chose it, fostering a personal connection with nature.

    This session cultivates a sense of wonder and connection to the larger environment. Students often report feeling calm or refreshed, illustrating the restorative power of mindful time in nature.

12. **Session S12 – Mindfulness for a Harmonious World:** The final session serves as a capstone, synthesizing the skills and insights gained throughout the program while emphasizing interconnectedness and responsible action. Students participate in a culminating group activity that visibly demonstrates interconnection. For example, they may sit in a circle and pass around a ball of yarn, with each student holding a part of the thread, creating a web that links everyone together. This "web of connection" exercise shows how each person is part of a connected community, where pulling one part of the yarn affects the entire web. The instructor relates this to the idea that our actions influence others and the world around us.

    Following this activity, students share reflections on their learning experiences and express

personal commitments to continue practicing mindfulness and kindness in their daily lives. They may complete sentences such as *"I am proud that I learned…"* or *"Going forward, I will practice…"* and share their responses with the class. By celebrating the completion of the program and reinforcing each student's role in fostering a more compassionate world, Session 12 consolidates the program's objectives and encourages students to apply their newfound skills beyond the classroom.

Throughout all sessions, instructors delivered content in a developmentally appropriate manner for two age groups: lower grades (1–3) and upper grades (4–6). The younger children's version of each session emphasized play-based learning, concrete examples, and simplified language, while the older children's version incorporated more complex discussion topics and reflection exercises to align with their higher cognitive abilities.

For instance, in the *Mindful Eating* session, lower-grade students engaged with a tangerine, focusing on its sensory qualities. In contrast, upper-grade students explored a dried persimmon and discussed the food's origin and the people involved in making it available. This tailored approach ensured that core concepts—such as gratitude in the case of mindful eating—were conveyed in an accessible way for younger students and in a more nuanced manner for older students.

These adaptations were consistently applied across all sessions, maintaining engagement and relevance for participants of different developmental stages. In summary, the 12-week MSEG program provided a structured yet flexible curriculum that integrated mindfulness practice with social-emotional learning. By utilizing developmentally tailored, experiential activities, the program fostered self-awareness, emotional regulation, compassion, and a sense of interconnectedness in elementary school students.
